# Supplementary material for: ArterialNet: Reconstructing Arterial Blood Pressure Waveform With Wearable Pulsatile Signals, a Cohort-Aware Approach
Source: IEEE Open J Eng Med Biol. 2025 Dec 1;7:14–9. doi: 10.1109/OJEMB.2025.3639174 (PMC12772991; doi:10.1109/OJEMB.2025.3639174)
Supplement: Supplementary Materials [file supp1-3639174.docx]

**Supplementary Materials**

ArterialNet: Reconstructing Arterial Blood Pressure Waveform with Wearable Pulsatile Signals, a Cohort-Aware Approach

Sicong Huang, *Student Member, IEEE*, Roozbeh Jafari, *Fellow, IEEE*, and Bobak J. Mortazavi, *Senior Member,* IEEE

## Data Processing

F

or MIMIC dataset, the PPG and ECG signals were filtered with a finite impulse response (FIR) bandpass filter of (0.5-8 Hz). Whereas the non-ICU Bio-Z pulsatile waveforms were preprocessed with a minimum-order, zero-phase, infinite impulse response (IIR) bandpass filter of (0.6-3 Hz) on both PPG and Bio-Z to remove artifacts while preserving phase relationships [1]. We then aligned pulsatile features with ABP waveforms on both datasets via phase shifting and segmented them into cardiac cycles using fiducial points [2, 3].

For MIMIC dataset, the PPG and ECG signals were filtered with a finite impulse response (FIR) bandpass filter of (0.5-8 Hz). Whereas the non-ICU Bio-Z pulsatile waveforms were preprocessed with a minimum-order, zero-phase, infinite impulse response (IIR) bandpass filter of (0.6-3 Hz) on both PPG and Bio-Z to remove artifacts while preserving phase relationships [1]. We then aligned pulsatile features with ABP waveforms on both datasets via phase shifting and segmented them into cardiac cycles using fiducial points [2, 3].

## Model Pretraining

We selected five patients who were normotensive at admission [27172, 47874, 94897, 56038, 82574] from our MIMIC-III cohort, by training on four subjects and validating on the last one during regularization. We performed thorough hyperparameter tuning and reported the tuning scope in our repository. Our best-performing pretraining model, ArterialNet, had a batch size of 512, a learning rate of 1e-5, a weight decay of 1e-2, and was trained for 75 epochs. Additionally, we observed that disabling the cohort-aware regularization for the first 10 epochs could accelerate model convergence.

## Analyzing Neural Network Layers

While ArterialNet as a multi-layered architecture achieved overperformance, understanding the contribution of individual components to overall improvement offers critical insights about each component’s importance. We performed a series of ablation studies on the MIMIC dataset to systematically mask or alter each component and measure changes in performance. We performed each experiment by disabling all ArterialNet components but the ablated one and compared the results against a barebone transformer (ArterialNet disabled) and full ArterialNet results (italicized) in Table 1.

Table 1: Ablation study of ArterialNet components on the MIMIC dataset. Experiments were performed by disabling all components but the ablated one.

| Ablation Type | Performance Metrics (RMSE and MAE in mmHg, no unit for Pearson’s R) | | | | | | | | | |
| --- | --- | --- | --- | --- | --- | --- | --- | --- | --- | --- |
|  | ABP (SD) | | | SBP (SD) | | | DBP (SD) | | | |
|  | RMSE | MAE | R | RMSE | MAE | R | RMSE | MAE | R |  |
| Waveform + gradient | 10.91 (1.14) | 5.97 (0.52) | 0.86 (0.02) | 11.32 (0.89) | 8.16 (0.64) | 0.85 (0.02) | 19.62 (5.13) | 17.08 (4.94) | 0.20 (0.11) |  |
| Waveform + morphologies | 10.03 (1.63) | 5.49 (0.48) | 0.88 (0.04) | 9.07 (2.09) | 7.57 (0.17) | 0.87 (0.00) | 9.12 (2.99) | 4.93 (1.31) | 0.41 (0.02) |  |
| Waveform + gradient + morphologies | 10.04 (2.06) | 6.86 (2.43) | 0.91 (0.02) | 11.64 (1.79) | 8.44 (1.40) | 0.78 (0.13) | 13.34 (3.75) | 8.79 (2.38) | 0.41 (0.03) |  |
| Waveform + correlation loss | 11.04 (3.06) | 6.86 (2.43) | 0.91 (0.00) | 11.64 (1.79) | 8.44 (1.40) | 0.78 (0.13) | 13.34 (6.75) | 8.79 (3.38) | 0.41 (0.03) |  |
| Waveform + alignment loss | 13.28 (1.48) | 8.44 (1.61) | 0.84 (0.03) | 16.30 (5.22) | 11.66 (3.45) | 0.55 (0.29) | 15.64 (8.42) | 11.75 (6.46) | 0.23 (0.12) |  |
| Waveform + correlation  + alignment loss | 9.95 (0.72) | 6.59 (0.65) | 0.93 (0.01) | 10.44 (1.24) | 7.28 (1.21) | 0.82 (0.02) | 6.92 (0.20) | 5.17 (0.28) | 0.65 (0.01) |  |
| Cohort-aware regularization | 9.20 (0.29) | 5.52 (0.53) | 0.91 (0.02) | 7.28 (0.81) | 5.92 (0.75) | 0.86 (0.01) | 9.01 (3.50) | 8.07 (4.39) | 0.65 (0.04) |  |
| Barebone transformer  (ArterialNet-less) | *10.31 (0.86)* | *6.11 (0.59)* | *0.89 (0.03)* | *11.40 (1.14)* | *8.17 (0.91)* | *0.74 (0.03)* | *18.32 (6.38)* | *12.58 (4.56)* | *0.28 (0.07)* |  |
| *No ablation*  *(ArterialNet)* | **5.41 (1.35)** | **4.17 (1.29)** | **0.91 (0.04)** | **5.26 (1.35)** | **4.15 (1.32)** | **0.90 (0.03)** | **4.01 (1.55)** | **3.17 (1.37)** | **0.88 (0.01)** |  |

### Feature Extractor

To derive more information of pulsatile signals, we extended our feature extractor by enabling it to compute and expand input dimensions using 1^st^ and 2^nd^ gradients of the original pulsatile signals. The results using this approach was reported in the “Gradient” row of Table 1. Comparing them against barebone results, we observed that the changes in performance were statistically insignificant.

The feature extractor could also exploit domain knowledges and rule-based features in addition to its automatic feature extraction. We achieved it by applying early fusion concatenation and feeding the unified embeddings to the downstream seq2seq backbone [4]. In our ablation, we computed 11 rule-based morphological features using previous work’s method and reported results in the “Morphologies” row of Table 1 [5]. Combining both features to feature extractor, we improved Pearson’s R of 0.02, 0.04, and 0.13 for ABP, SBP, and DBP, respectively.


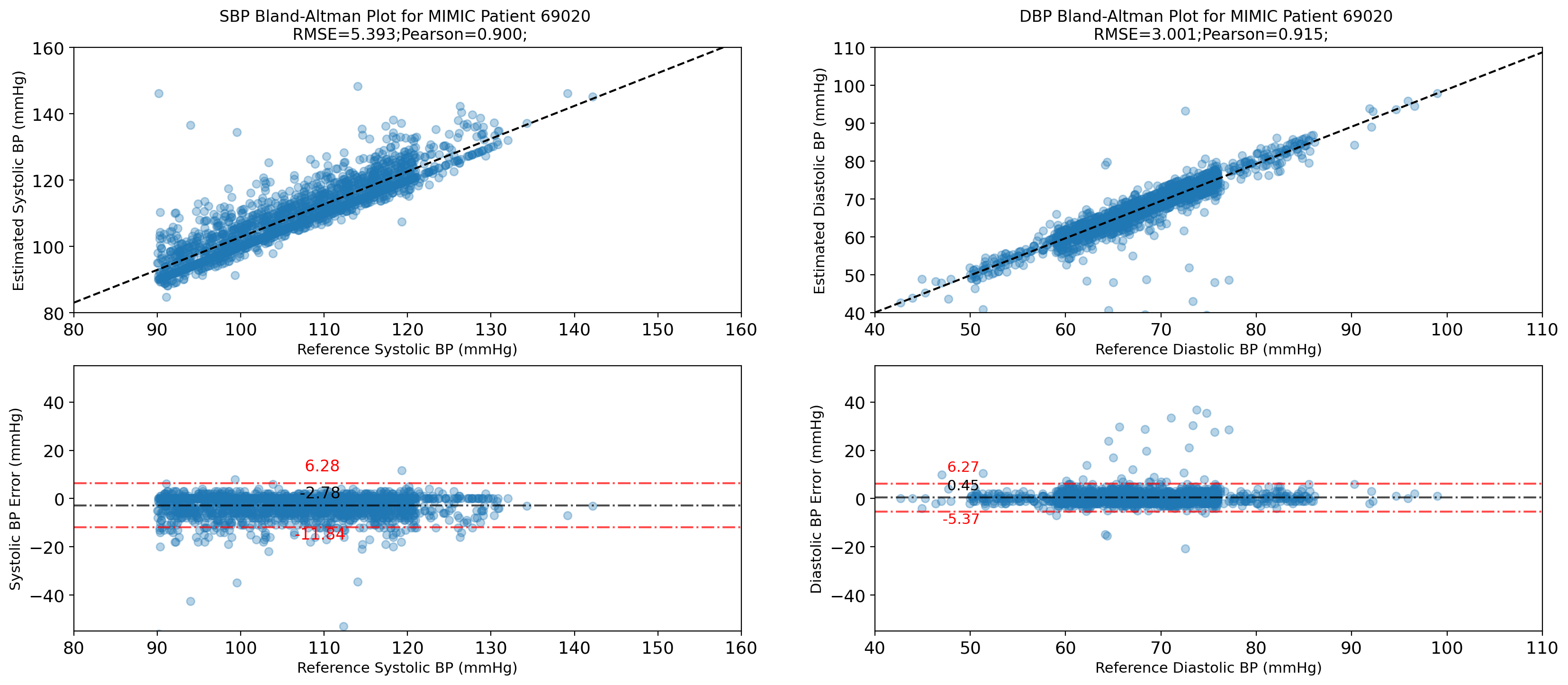


Figure 1: An example of Bland-Altman analysis on estimated systolic and diastolic blood pressure values derived ArterialNet’s ABP waveform

### Hybrid Objective Function

To understand the impact of each objective function on model performance, we iteratively evaluated the effect of adding correlation loss, alignment loss, and both alongside waveform loss. We included waveform loss through all experiments since the model required it to converge. We reported the results in rows 4~6 of Table 1. We observed improvements in Pearson’s R of 0.04, 0.08, and 0.37 for ABP, SBP, and DBP, respectively when all three losses were used.

### Cohort-Aware Regularization

All ablation studies above were trained from scratch, without invoking finetuning paradigm to ensure fairness versus the barebone baseline. Conversely, since cohort-aware regularization was designed specifically for the pretraining stage, we leveraged ArterialNet’s training paradigm (disabling hybrid objectives disabled and using original feature extractor) to complete the ablation experiment. Thus, the ablation results could also serve as comparison of our two-stage paradigm vs. training from scratch. With results reported on “Cohort-aware regularization row” of Table 1, we observed improvements in Pearson’s R of 0.02, 0.12, and 0.37 for ABP, SBP, and DBP, respectively.

References

[1] T. Athaya, and S. Choi, “A Review of Noninvasive Methodologies to Estimate the Blood Pressure Waveform,” *Sensors (Basel),* vol. 22, no. 10, May 23, 2022.

[2] Z. Nowroozilarki, B. J. Mortazavi, and R. Jafari, “Variational autoencoders for biomedical signal morphology clustering and noise detection,” *IEEE Journal of Biomedical and Health Informatics*, 2023.

[3] B. Ibrahim, and R. Jafari, “Cuffless blood pressure monitoring from an array of wrist bio-impedance sensors using subject-specific regression models: Proof of concept,” *IEEE transactions on biomedical circuits and systems,* vol. 13, no. 6, pp. 1723–1735, 2019.

[4] S. N. Shukla, and B. M. Marlin, “Integrating physiological time series and clinical notes with deep learning for improved icu mortality prediction,” *arXiv preprint arXiv:2003.11059*, 2020.

[5] J. Martinez, Z. Nowroozilarki, R. Jafari, and B. J. Mortazavi, “Data-driven guided attention for analysis of physiological waveforms with deep learning,” *IEEE Journal of Biomedical and Health Informatics,* vol. 26, no. 11, pp. 5482–5493, 2022.
